# Recruitment-Potential-Oriented Mechanical Ventilation Protocol and Narrative Review for Patients with Acute Respiratory Distress Syndrome

**DOI:** 10.3390/jpm14080779

**Published:** 2024-07-23

**Authors:** Chieh-Jen Wang, I-Ting Wang, Chao-Hsien Chen, Yen-Hsiang Tang, Hsin-Wei Lin, Chang-Yi Lin, Chien-Liang Wu

**Affiliations:** 1 Division of Pulmonary and Critical Care Medicine, Department of Internal Medicine, MacKay Memorial Hospital, Taipei 104217, Taiwan; lce@mmh.org.tw (C.-Y.L.); wuchienliang.4004@mmh.org.tw (C.-L.W.); 2 Department of Medicine, MacKay Medical College, New Taipei City 25245, Taiwan; cherrywang822.5105@mmh.org.tw (I.-T.W.); tang.4131@mmh.org.tw (Y.-H.T.); 3 Department of Critical Care Medicine, MacKay Memorial Hospital, Taipei 104217, Taiwan; 4 Division of Pulmonary and Critical Care Medicine, Department of Internal Medicine, Taitung MacKay Memorial Hospital, Taitung 950408, Taiwan; 5 Department of Critical Care Medicine, MacKay Memorial Hospital, Tamsui 251020, Taiwan; 6 Department of Chest Medicine, Taoyuan General Hospital, Ministry of Health and Welfare, Taoyuan 33004, Taiwan; 05615@mail.tygh.gov.tw

**Keywords:** acute respiratory failure, APRV, ARDS, ECMO, mechanical ventilation, nitric oxide, PEEP, pressure–volume curve, recruitment, protocol

## Abstract

Even though much progress has been made to improve clinical outcomes, acute respiratory distress syndrome (ARDS) remains a significant cause of acute respiratory failure. Protective mechanical ventilation is the backbone of supportive care for these patients; however, there are still many unresolved issues in its setting. The primary goal of mechanical ventilation is to improve oxygenation and ventilation. The use of positive pressure, especially positive end-expiratory pressure (PEEP), is mandatory in this approach. However, PEEP is a double-edged sword. How to safely set positive end-inspiratory pressure has long been elusive to clinicians. We hereby propose a pressure–volume curve measurement-based method to assess whether injured lungs are recruitable in order to set an appropriate PEEP. For the most severe form of ARDS, extracorporeal membrane oxygenation (ECMO) is considered as the salvage therapy. However, the high level of medical resources required and associated complications make its use in patients with severe ARDS controversial. Our proposed protocol also attempts to propose how to improve patient outcomes by balancing the possible overuse of resources with minimizing patient harm due to dangerous ventilator settings. A recruitment-potential-oriented evaluation-based protocol can effectively stabilize hypoxemic conditions quickly and screen out truly serious patients.

## 1. Introduction

Acute respiratory distress syndrome (ARDS) was initially described by Ashbaugh et al. in 1967 [1]. It is characterized by inflammatory injury to alveolar cells, which form the barrier of the microscopic air sacs of the lungs. Increased alveolar–capillary permeability to liquid, proteins, neutrophils, and red blood cells leads to surfactant dysfunction, activation of the immune system, and dysregulation of the body’s blood clotting, resulting in severe hypoxemia [2]. In the past decades, the focus of mechanical ventilation strategies in patients with ARDS has progressed from maintaining adequate oxygenation and eliminating carbon dioxide to preventing ventilator-associated lung injury (VALI) [2,3]. Considerable advancements including small tidal volume [4] and prone position [5] have been made. However, issues such as the high/low positive end-expiratory pressure (PEEP) strategy [6,7,8,9], the recruitment maneuver (RM) [8,10,11,12,13], neuromuscular blockade [14,15], extracorporeal membrane oxygenation (ECMO) [16,17], airway pressure release ventilation (APRV) [18,19], inhaled nitric oxide (iNO) [20,21,22], and high-frequency oscillatory ventilation [23,24,25] persist. The goal of this article is to integrate these known-to-be effective and potentially effective concepts to provide clinicians with quick and time-saving guidelines for mechanical ventilator settings in patients with ARDS.

## 2. Recognize That the Patient Has ARDS

The first step is to recognize whether the patient’s lung is recruitable. According to the LUNGSAFE study [26], up to 50% of patients with mild ARDS and 20% of those with severe ARDS were not recognized in a real-life setting. Clinical vigilance is still the most important prerequisite for clinicians. With the understanding that mechanical ventilation itself can induce and enhance lung injury, limiting tidal volume [27] and inspiratory pressure [28] in all mechanically ventilated patients is mandatory. When patients cannot be ventilated safely [6] and/or achieve safe oxygenation goals (defined as PaO_2_ 55–80 mmHg or SpO_2_ 88–95%) [4], we strongly recommend prompt evaluation of their lung recruitment potential.

## 3. Choose the Appropriate PEEP

### 3.1. General Consideration

PEEP (5–20 cm H_2_O) is a key element of mechanical ventilation in all patients with ARDS to improve oxygenation and maintain alveolar recruitment [29]. Unfortunately, there is still no ideal method to determine the optimal PEEP levels in individual patients [2]. Several studies have suggested that higher PEEP strategies resulted in higher oxygenation but did not provide superior patient-centered outcomes [6,7,8,30]. However, a meta-analysis [9] of these trials suggested that higher PEEP levels might be preferable in patients with moderate or severe ARDS. Gattinoni et al. [31] also reported that the percentage of potentially recruitable lung in patients with ARDS is extremely variable and strongly associated with the response to the PEEP. When the PEEP is too low, the patient is at risk of ventilator-induced lung injury from the cyclic opening and closing of the alveoli, whereas if the PEEP is too high, the patient may suffer from the risk of alveolar overdistension. The best way to avoid these scenarios is to evaluate patient lung recruitability. There are several methods present for this purpose, each with its own advantages and disadvantages (Table 1). From a clinician’s perspective, the most practical method is one that requires no or minimal special equipment, is relatively simple and rapid to perform, and yields a clear result. PEEP INview [32,33] or electrical impedance tomography [34,35] is frequently mentioned in lung recruitability assessment, but its application relies on specific equipment and is time-consuming. Esophageal pressure-guided PEEP also requires special equipment and personnel training and can improve patient oxygenation [7,36] but not patient outcomes [36]. The evidence for its physiological goals is also questionable [37]. PEEP/FIO_2_ tables, first proposed by Brower et al. [4], have been created to guide clinicians [38], but randomized trials of PEEP titration using the low versus high PEEP/FIO_2_ tables have not demonstrated the superiority of either table [39]. The weakness of this approach is that its settings are based on consensus research rather than physiological evidence [6]. The recruitment-to-inflation ratio (R/I) method [40] performed by abruptly releasing PEEP (from 15 to 5 cm H_2_O) increases the expired volume: the difference between this volume and the volume predicted by compliance at low PEEP estimated the recruited volume by PEEP. R/I values ≥ 0.5 suggest more potential for lung recruitment with respect to lung inflation. However, this method can only provide limited information within a certain PEEP range, and several measurements are necessary if detailed adjustments are required [41]. It is time-consuming and involves relatively cumbersome procedures without direct targets. Lung ultrasound (LUS) can be useful for assessing the effectiveness of a recruitment maneuver [42] via morphological assessment [43] or score system [44], but these attempts are limited to clinical observations and not widely accepted standards.

Therefore, we recommend using the quasi-static respiratory system pressure–volume (P-V) curve to evaluate the recruitment potential of the patient’s lungs [45], which may allow for the individualization of initial PEEP settings in patients with ARDS. 

### 3.2. How to Perform P-V Curve and PEEP Titration

The procedure of P-V curve measurement is briefly described below (using a Hamilton^®^ ventilator in most scenarios (Hamilton Medical AG, Bondaduz, Switzerland)):

Sedate patients;
Use succinylcholine 1 mg/kg IV and repeat until the breathing has been controlled;
Always start from 0 cm H_2_O, targeting 35–40 cm H_2_O;
Hold at the top for 5–15 s and then passively exhale;
Pressure ramp: 3 cm H_2_O.

A lower inflection point (LIP) is defined as the pressure at the intersection of two lines: a low-compliance region at a low lung volume and a higher-compliance region at a higher lung volume on the lung P-V curve. The LIP indicates the pressure at which to prevent alveolar recollapse because it is the point of significant compliance change [46]. Once determined, the PEEP level should be started at a value of LIP + 2 cm H_2_O combined with a low tidal volume to provide lung volume recruitment and avoid overdistention. Further PEEP titration along the inspiratory limb is allowed if the physician considers that a higher PEEP level may be beneficial for the patient. In this scenario, Millington et al. [47] suggested calculating the driving pressure (DP) at different PEEP levels. Assuming the tidal volume does not change between the different PEEP levels (in this scenario, volume-targeted ventilation mode is preferred to make the calculation easier), the change in the DP can only be explained by a change in compliance. If the DP decreases at a higher level of PEEP, the higher PEEP trial can be considered successful. If the DP does not change, optimal compliance has potentially been achieved. However, a further increase in PEEP may still be safe if the plateau pressure limit is not violated. If the DP increases, then compliance has worsened, possibly indicating overdistension, and consideration should be given to lowering the PEEP. Compared with the R/I method, the single P-V curve measurement from zero end-expiratory positive pressure requires sedating and paralyzing patients, which may seem more cumbersome at first glance, but its measurement speed is rapid (usually less than one minute). In addition, a single measurement can provide sufficient information for PEEP titration, and patient sedation and paralysis are not mandatory. Two elastic P-V curve measurements along the tidal volume and starting from higher and lower PEEP are required. It only provides a rough range showing that higher PEEP may recruit more volume, but no warning of overdistension and multiple measurements at different PEEP is required for detail setting. Furthermore, the initially selected high PEEP (empirical assumption) may induce alveolar overdistension in less recruitable lungs; therefore, this R/I method is not recommended to evaluate recruitment potential because it is time-consuming, has unclear goals, and may cause harm.

### 3.3. Alternative Approaches

If it is not possible to calculate the P-V curve, the PEEP/FiO_2_ table [38] can be applied, modified with stress index measurement [48,49]. An alternative approach in this situation is to maintain spontaneous breathing in the patient to induce lung recruitment and reduce global stress/strain. APRV represents a pressure-limited time-cycled ventilator mode [19]. The potential benefits of APRV are related to spontaneous breathing and include the following: (1) better patient–ventilator synchrony and improved patient comfort [18,19]; (2) improvement in ventilation/perfusion matching by promoting more gas distribution to the nondependent lung regions [50]; and (3) potential decreases in sedation, analgesia, and neuromuscular blockade [19]. Zhou et al. [18] reported that early application of APRV in patients with ARDS improved oxygenation and respiratory system compliance, decreased Pplat, and reduced the duration of both mechanical ventilation and ICU stay. In our protocol, when the patient’s condition fulfills the criteria of a mild-to-moderate degree of ARDS, the clinician can choose to use APRV first. These two approaches (P-V curve measurement and APRV) are interchangeable and depend on the clinician’s preference.

### 3.4. Paralysis Strategy

Haren et al. reported that spontaneous breathing is common in patients with ARDS during the first 48 h of mechanical ventilation and is not associated with worse outcomes [51]. However, patients with injured lungs usually have a high respiratory drive due to gas exchange and respiratory mechanics’ impairment [52]. High respiratory drive results in strong inspiratory efforts, which may be accompanied by physiological effects (risk of overdistension, pendelluft, atelectrauma, and increase in vascular transmural pressure) [52] and impaired patient–ventilator interaction [53], leading to patient self-inflicted lung injury (P-SILI) [54]. If maintaining spontaneous breathing is desired, excess patient efforts are monitored by the Bertoni method [55], which uses the swing in the airway pressure generated by respiratory muscle effort under assisted ventilation when the airway is briefly occluded. It can be used to detect potential injurious inspiratory effort and dynamic lung stress. Although the early use of muscle relaxants to improve the ARDS prognosis is still controversial [14,15], we paralyze patients when they are diagnosed with severe acute respiratory distress syndrome or the patient’s breathing is deemed too vigorous, risking P-SILI.

## 4. Integrated Multiple Modalities for Severe Hypoxia: Prone, iNO, RM, and ECMO

### 4.1. Recruitment Maneuver

In patients assigned for P-V curve measurement, if a LIP is present, the patients are managed with PEEP titration first with or without prone positioning (as discussed in the following paragraph), even if the oxygenation goal has not been achieved. We do not routinely perform the RM because of a lack of efficacy [12], a higher rate of complications [56], and the possibility of causing harm [13]. If the RM is chosen by clinicians, we suggest the target would be PaO_2_/FiO_2_ ≥ 350 [57] or index PaO_2_ + PaCO_2_ ≥ 400 [58]. The RM would then be repeated every 8 h for 2 days, and the PEEP would be set at 2 cm H_2_O above the derecruitment point on the deflation limb of the P-V curve [8] after each RM.

### 4.2. Meticulous PEEP Titration

If a LIP is not detectable, prone positioning and inhaled vessel dilators (mainly iNO) may be tried before considering ECMO. However, the absence of a LIP may not suggest the lack of recruitment potential because lung recruitment is a time-dependent phenomenon that cannot be described by a single P-V curve [59]. In this situation, we consider whether (a) there is volume increase of 2 mL/kg ideal body weight (or >200 mL) at the end of pressure holding during the P-V curve measurement [45]; or (b) the shape of the inspiration limb of the P-V curve is convex and/or the volume difference between the inflation and deflation curve at 20 cm H_2_O is >500 mL [45]; or (c) the normalized maximal distance% is >41%. The normalized maximal distance% represents the maximal delta volume between the inflation and deflation limbs of the P-V curve divided by the maximum volume of the P-V curve measurement [60]. If a patient’s P-V curve does not have a LIP but has any of these three factors, prudent PEEP titration as previously described [47] is still possible.

### 4.3. iNO

In patients who are considered to have poor recruitment potential or PEEP titration not yielding satisfactory results or reached the upper pressure limit of safety [6], we recommend using iNO, prone positioning, or their combination. Although iNO has not been shown to reduce mortality in patients with ARDS in past studies [20,21], transient improvements in oxygenation may be possible [22], which may allow time for the underlying problems to respond to definite treatments or rescue interventions [61,62]. We strongly recommend the application of iNO if it is available, and there are no contraindications [63] in this situation.

### 4.4. Prone

In most situations, the patients require prone positioning. The prone position (PP) has been used since the 1970s to treat severe hypoxemia in patients with ARDS, but its efficacy in reducing mortality was only confirmed with the publication of the landmark PROSEVA trial [5]. Although lung recruitment induced by PP has been suggested by some authors [64,65,66] to be an important mechanism in improving oxygenation, it is not the only mechanism [67,68].

Pelosi et al. [69] even found that improved oxygenation after PP often occurred without corresponding improvements in compliance of the respiratory system, and that the presence or lack of a LIP could predict PP response. Considering prone positioning is not without risks [70,71] and turning patients requires a coordinated team effort, we recommend that prone positioning be applied to patients with refractory hypoxemic ARDS, but that it is better to start after evaluating the recruitment potential. The optimal duration of each PP session remains uncertain, but keeping the PP for >16 h/day [5] is used in most institutes. We follow the same policy and usually keep patients in the PP until the next morning. We turn them around to evaluate whether they should continue to remain prone and perform other necessary examinations and treatments. During the COVID-19 pandemic, some centers independently reported extending prone positioning beyond 24 h [72,73]. Most reported maintaining patients in the prone position until significant clinical improvement was achieved. There are two main considerations for extending the prone duration: one is the lack of human resources, and the other is the clinical effect. Douglas et al. [74] maintained PP sessions in patients with COVID-19-related ARDS until the following criteria were reached: P/F ratio > 150 with FiO_2_ > 60% and PEEP levels < 10 cm d’H_2_O. This protocol led to sessions of a median duration of 2.95 days among survivors and 3.3 days among nonsurvivors. Considering that there are some complications associated with PP that may increase over time, like pressure sores, regurgitation, and brachial plexus palsy [72], we believe that the current evidence is not sufficient to support the routine use of extended PP for patients with severe ARDS.

### 4.5. ECMO

Since patients who develop the most severe forms of ARDS have mortality rates exceeding 50% despite optimal supportive care [75], ECMO is frequently considered as the last resort. After the CESAR [16] and EOLIA [17] trials were published, the early application of ECMO for patients with severe ARDS became a hot topic among the experts. We admit that some patients are not suitable for step-by-step adjustment and need prompt rescue intervention. Also, even if patients are adequately ventilated, the ventilator setting may cause damage; thus, the patient still cannot survive ARDS. In this scenario, using ECMO to rest the lungs may improve patient survival. Because late crossover may be futile [17,75], the decision to start ECMO should be evaluated early. Evaluating the RESP and PRESERVE scores [76,77] may help clinicians to judge whether to continue current management, switch to palliative care, or consider placing the patient on ECMO. Whether a prone trial should be tried before starting ECMO is open to the clinician’s judgment [78]. In our protocol, we perform arterial blood gas analysis and monitor respiratory mechanics every 2 to 4 h to determine whether the patient’s ventilation meets oxygenation goals and remains within safe physiological pressure limits. All management procedures can be completed within 8–24 h before they are considered to be ineffective or harmful. For patients with severe hypoxia (P/F ratio < 60 mg > 2 h, or P/F < 80 mmHg for 6 h, or PaCO_2_ > 60 mmHg plus PH < 7.1) [17], fast track to ECMO support is allowed at the request of the in-charge physician. In patients who can maintain adequate oxygenation using traditional methods but have inadequate ventilation (PaCO_2_ > 60 mmHg plus PH < 7.1), ECMO (or ECCO2R) is also an option for ultraprotective low tidal volume mechanical ventilation [79,80,81].

## 5. Lung Mechanics Safety Check

If the patients become stable at any stage of the protocol (before ECMO application), right ventricle protection is considered to avoid acute cor pulmonale [82]. Airway pressure changes due to the fact that PEEP induces a series of alterations in the right ventricle size and in pulmonary circulation that can lead to right heart hemodynamic impairment. We try to minimize lung stress by limiting the plateau pressure to <25–28 cm H_2_O and driving pressure to <15 cm H_2_O [83]. In addition, arterial carbon dioxide concentration is strictly maintained to <60 mmHg. Prone positioning is also considered for this purpose (if not yet applied). The possibility of overdistension is also evaluated via PEEP INview [32] or electrical impedance tomography [34] if the patient can be safely ventilated with FiO_2_ < 60% at a PEEP value > LIP + 2.

**Figure 1:**
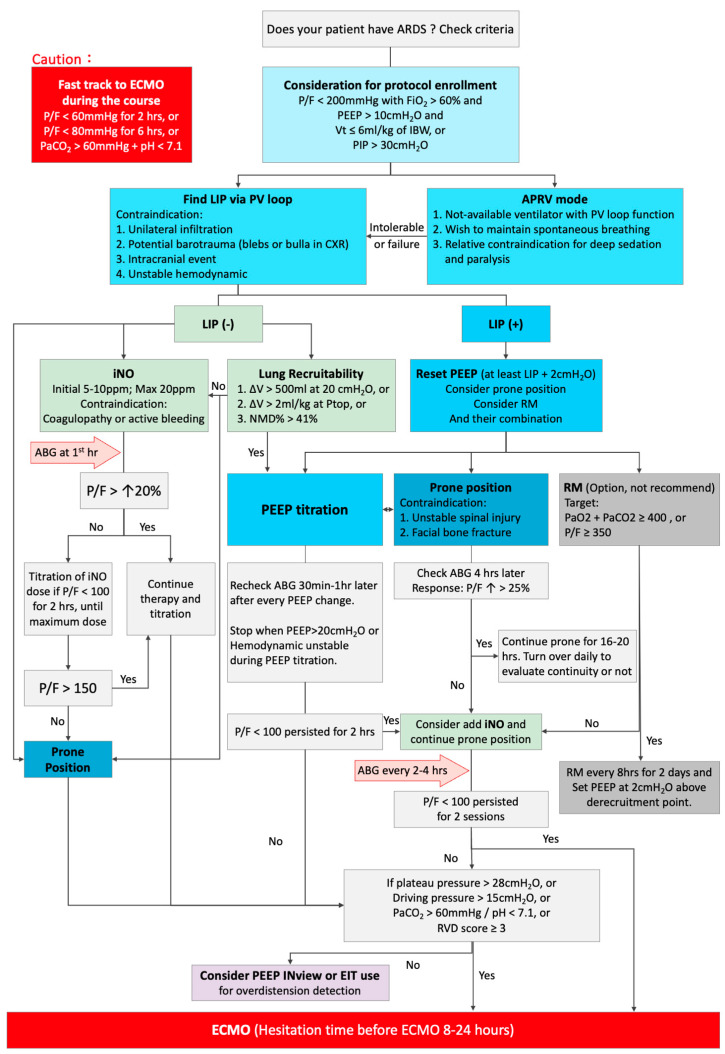
The flowchart of the protocol in a patient with acute respiratory distress syndrome (ARDS). ABG: arterial blood gas, APRV: airway pressure release ventilation, ECMO: extracorporeal membrane oxygenation, EIT: electrical impedance tomography, hrs: hours, IBW: ideal body weight, iNO: inhaled nitric oxide, LIP: lower inflection point, NMD: normalized maximum distance between the inflation and deflation limb of pressure volume curve, PEEP: positive end-expiratory pressure, P/F: PaO_2_/FiO_2_, PIP: peak inspiratory pressure, Ptop: holding at 35–40 cm H_2_O for 5–15 s, PV loop: quasi-static respiratory system pressure–volume loop, RVD: right ventricular dysfunction, RM: recruitment maneuver, ∆V: volume difference between inflation and deflation limb, ↑: increased.

When the pressure limit (plateau/driving pressure) is exceeded [4,84] and/or the right ventricular dysfunction (RVD) score [83] is ≥3 despite achieving adequate oxygenation and ventilation via traditional mechanical ventilator settings, ECMO would be considered to minimize VALI [2,3,79,81]. A summary of our proposed protocol is shown in Figure 1.

## 6. Conclusions

In summary, our proposed protocol offers a structured, timely, and resource-saving approach for patients with severe hypoxic respiratory failure. The concept of the protocol is based on whether the patient’s lungs are likely to be recruited or not. The optimal positive end-expiratory pressure (PEEP) in mechanical ventilation for patients with acute respiratory distress syndrome (ARDS) is controversial. The concept of “Baby lung” [85] has convinced many clinicians that positive pressure application should be limited to avoid ventilator-associated lung injury (VALI). Research conducted in the early 2000s demonstrated that using small tidal volumes [4] can improve the prognosis of patients with ARDS and increase the number of ventilator-free days, supporting this approach. However, some experts contend that employing appropriate or higher-than-normal pressure for ventilation, a strategy known as a recruitment maneuver (RM), can restore lung volume in patients with ARDS [8,10,11]. These divergent strategies both present benefits and challenges. When attempting to employ the standard approach by limiting the application of positive pressure, clinicians often encounter situations where they cannot adequately ventilate patients, necessitating alternative measures such as causing the patients to inhale nitric oxide, assume a prone position, receive high-frequency oscillatory ventilation, or receive extracorporeal membrane oxygenation (ECMO). These adjunctive techniques employ considerable equipment and human resources, and not all hospitals have the relevant capabilities. In contrast, when applying an RM, although the lung capacity and oxygenation of some patients temporarily improve, other patients may experience complications related to the RM, including mortality [13]. Even with improved oxygenation, patients receiving RMs may not experience improved survival. The optimal positive pressure likely lies between the extremes. The reason for these divergent results is that ARDS is a clinical diagnosis. Whether the oxygenation of patients with similar presentation can be improved through positive pressure cannot be easily judged. There remains no consensus regarding the efficacy of high versus low PEEP strategies [6,9]. The most effective approach to resolving this controversy is a determination of whether a patient’s lungs are recruitable. We choose the P-V curve [45] but not PEEP INview [32], esophageal pressure guidance [7], R/I ratio method [40], or electrical impedance tomography [34] for the initial evaluation because the former method is easier to perform and provides results quickly. However, there are several concerns when interpreting the P-V curve. First, there is no standard method for acquiring P-V curves [86,87]. Second, significant inter- and intra-observer variability in identifying the LIP in P-V curves obtained from patients with ARDS has been noted, with maximum differences of up to 11 cm H_2_O between observers for the same patient’s curve [88,89]. In a uniformly recruited lung, the LIP should be well defined and sharp [90]; however, in nonuniform lung recruitment, alveoli are recruited across a broader range of pressures, leading to an absent or unclear LIP [91]. To overcome this limitation, we use other methods to investigate the recruitment potential [45,47,60]. In addition, there is a paucity of data showing benefits in morbidity and mortality with the use of P-V curves [92]. Our recruitment-potential-oriented ventilator setting does not seek to optimize ventilation but to minimize VALI. The basis of this protocol is adequate oxygenation without excessive stress or strain. Following the publication of the results of the CESAR [16] and EOLIA [17] trials, the early application of ECMO for patients with severe ARDS has been the subject of fierce debate. We acknowledge that some patients are unsuitable for incremental adjustments but require an immediate rescue intervention. We also recognize that even when patients can be adequately ventilated, the ventilator settings may cause harm, resulting in death. In such cases, using ECMO to rest the lungs may enhance the likelihood of patient survival. In summary, our proposed protocol offers a personalized strategy for patients with ARDS that minimizes several of the drawbacks of other approaches.

## Figures and Tables

**Table 1:** Lung recruitment potential evaluation tools.

Method	Advantage	Disadvantage
PEEP/FiO_2_ table [4,38]	No specific equipment requirement Expert consensus with a lot of experience	Lack of physiologic evidence Risks of overdistension
PEEPIN view [32,33]	Best compliance achievement Low overdistension risk Minimal sedation requirement	Dedicated equipment requirement Long duration of measurement Needs manual setting of measurement range
EIT [34,35]	Optimal compliance achieved Minimal overdistension risk Minimal sedation request	Dedicated equipment requirement Offline analysis Long duration of measurement
Esophageal pressure-guided PEEP setting [7,36]	Specific target presence Better oxygenation	Limited physiologic evidence Specific equipment requirement Specific personal training requirement
Recruitment-to-inflation ratio method [40,41]	Minimal sedation requirement Allows fine-tuning of PEEP settings via multiple steps of measurements	Limited evidence Long duration of measurement Specific equipment requirement Needs manual setting of measurement range
Lung ultrasound [42,43,44]	Minimal equipment requirement Additional hemodynamic monitoring capabilities	Limited evidence Labor-intensive task Specific personal training requirement
P-V curve [45]	Able to quickly achieve an initial stable state Specific target presence Allows multiple fine-tuning in one measure	Specific equipment requirement Inter- and intra-observer variation Needs auxiliary measurement to avoid overdistension Needs sedation and paralysis

## Data Availability

Data are contained within the article.
